# Hormone-Mediated Gene Regulation and Bioinformatics: Learning One from the Other

**DOI:** 10.1371/journal.pone.0000481

**Published:** 2007-05-30

**Authors:** João Carlos Sousa, Manuel João Costa, Joana Almeida Palha

**Affiliations:** Life and Health Sciences Research Institute (ICVS), School of Health Sciences, University of Minho, Campus Gualtar, Braga, Portugal; University of the Western Cape, South Africa

## Abstract

The ability to manage the constantly growing clinically relevant information in genetics available on the internet is becoming crucial in medical practice. Therefore, training students in teaching environments that develop bioinformatics skills is a particular challenge to medical schools. We present here an instructional approach that potentiates learning of hormone/vitamin mechanisms of action in gene regulation with the acquisition and practice of bioinformatics skills. The activity is integrated within the study of the Endocrine System module. Given a nucleotide sequence of a hormone or vitamin-response element, students use internet databases and tools to find the gene to which it belongs. Subsequently, students search how the corresponding hormone/vitamin influences the expression of that particular gene and how a dysfunctional interaction might cause disease. This activity was presented for four consecutive years to cohorts of 50–60 students/year enrolled in the 2^nd^ year of the medical degree. 90% of the students developed a better understanding of the usefulness of bioinformatics and 98% intend to use web-based resources in the future. Since hormones and vitamins regulate genes of all body organ systems, this activity successfully integrates the whole body physiology of the medical curriculum.

## Introduction

The knowledge accumulated and the technological evolution in the field of genetics has grown immensely over the last years. In accordance, the wealth of genetics education resources available is presently considerable [1]. Reflecting the importance of genetics and of genetic databases and bioinformatics tools, some medical schools have addressed recent recommendations of incorporating bioinformatics in the medical curriculum [2]. Examples of such include the implementation of short introductory modules to online resources such as the National Center for Biotechnology information (NCBI) or the Online Mendelian Inheritance in Man (OMIM), followed by queries to identify genes and mutations associated with diseases [3], [4]. Data have been gathered demonstrating that medical students develop a positive impression from hands-on bioinformatics learning sessions and that some students continue to use the resources later in their clinical training [4]. By having students work under the context of clinical vignettes, the published educational approaches associate bioinformatics with clinically relevant subjects [3], [4]. By asking students to do queries, the benefits of active learning are invoked. However, these approaches are mostly isolated in the curriculum. In addition, by usually focusing on a single gene, the learning investment lies on the straightforward use of the bioinformatics resources to find a “correct solution”, which might be distant from real clinical scenarios in medical practice. As a consequence, students might miss opportunities to develop, on their own, an impression on the multitude of factors and diseases as well as on some of the web/bioinformatics resources specificities.

Nowadays, the interplay between genes and environment in disease etiology and response to therapy are well recognized. Hormones and vitamins are key modulators of gene expression that should be considered. The existence of single nucleotide polymorphisms in the promoter of genes whose expression is regulated by hormones and vitamins can alter the binding of transcription factors and, therefore, influence their expression. Most recent research on this field is taking advantage of knowledge on the human genome sequence through the use of bioinformatics tools to predict and analyze changes in transcriptional regulation induced by drugs or environmental insults [5]. Importantly, the interaction between hormones/vitamins, genes, environment, and the usage of bioinformatics and molecular biology tools is predicted to become one of the most important subjects in clinical practice in the near future [6], [7].

Therefore, bringing regulation of gene expression into play can be further explored in the educational context using bioinformatics tools. We present here an instructional approach that promotes the learning of hormone/vitamin mechanisms of action in gene regulation with the acquisition and practice of bioinformatics skills.

## Analysis

### Integration in the medical curriculum

**Figure 1:**
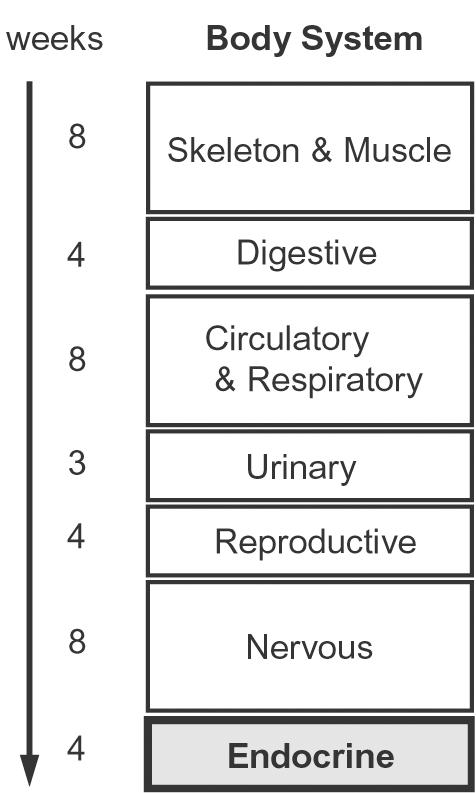
Chronological organization of the Functional Organ Systems curricular area.

This activity has been presented to 2^nd^ year undergraduates of the 6 year-long medical curriculum at the School of Health Sciences, University of Minho. It is a component of the curricular unit “Functional and Organic Systems” which integrates the perspectives of anatomy, biochemistry, embryology, histology and physiology. Previously, students had been introduced to essential molecular and cellular biology concepts like gene, promoter, or transcription factor. Chronologically, the activity we present here is offered in the last of the organ systems studied, the Endocrine, after students had gone through other body systems (figure 1). In this way, the interplay between the mechanisms of hormone action and the organ systems physiology, and the consequences of hormone gene regulation is easily recognized by students. The activity requires that students make extensive use of the internet but no specific previous training on bioinformatics is provided.

### Instructional and educational aims

The instructional outcomes are designed for the students to: 1) understand how hormones and vitamins regulate gene expression; 2) recognize bioinformatics as an essential clinical genetics asset for a physician; 3) perceive the usefulness of online resources and 4) develop basic bioinformatics query skills. The approach also targets the following educational objectives: 1) promote student-centered learning, by providing opportunities for self-paced discovery; 2) favor deep learning by anchoring essential concepts on hormone/vitamin response mechanisms in contexts or experiences of relevance in medicine; 3) promote teamwork.

### Design and requirements

The activity comprises 16 h distributed in 7 sessions (Table 1). It takes place throughout a 4 week-long endocrine module that covers other aspects of anatomy, biochemistry, embryology, histology and physiology. Students are randomly assigned to groups of up to 5, within classes of 30. The classroom is equipped with one computer with internet access per student. One member of the teaching staff, from now on referred to as instructor, supervises all the activity.

**Table 1:** Activity syllabus and instructor and students role during the sessions.

Session	Description	Time (h)	Instructor	Students
1	Activity presentation	1	Active role	All
2	Bioinformatics tools exploration	3	Available to discuss specific aspects	Groups
3	Usage of tools to find gene and hormone/receptor	2		Groups
4	Literature database search	2		Groups
5	Written report preparation	2	Monitoring	Groups
6	Presentation preparation	2	Absent	Groups
7	Group work presentation	4	Audience	All

#### Session 1 (1 h): Explaining the activity, launching the challenge

The first session defines the general guidelines, goals and working plan. The instructor introduces the activity by generating a discussion on how hormones and vitamins might relate to disease through gene regulation and in a given environmental context. The challenge is then launched. Each group of students is given a short DNA sequence (containing the response element for a specific hormone/vitamin nuclear receptor) that will be used as the starting point for the group assignments: *i*) identify the hormone response element consensus sequence and variations to the consensus sequence; *ii*) relate the hormonal regulation of the particular gene with its function from the cellular to the body system's level, in health and disease; *iii*) annotate all the decisions on where and how to search for information using bioinformatics tools, as it occurs over time; *iv*) write a report in the format of a scientific journal, restricted to a total of 2000 words and prepare, based on this, a 6 minutes oral presentation.

The bioinformatics resources necessary to carry out the activity are summarized in Table 2. The usefulness of these tools has been extensively described by other authors [8], [9]. Instead of thoroughly presenting the tools, students are orientated to online tutorials. Since there are several possible strategies to successfully accomplish the task, it becomes essential that students discover and explore, by themselves, how to use each of the tools. Since no precise directions are given, students are encouraged to develop team work strategies, distribute responsibilities and accurately and appropriately annotate the information gathered.

**Table 2:** List of bioinformatics tools and databases students used.

Database/Tool	url
PubMed	http://www.pubmed.gov
ISI Web of Science	http://portal.isiknowledge.com
Google Scholar	http://scholar.google.com
TRANFAC	http://www.gene-regulation.com/
Blast	http://www.ncbi.nlm.nih.gov/BLAST/
JASPAR	http://jaspar.genereg.net/
OMIM	http://www.ncbi.nlm.nih.gov/omim/
Genbank	http://www.ncbi.nlm.nih.gov/entrez/query.fcgi?db = Nucleotide
National Center for Biotechnology Information	http://www.ncbi.nlm.nih.gov/
European Bioinformatics Institute	http://www.ebi.ac.uk/Tools/

#### Sessions 2–6 (11 h): addressing the challenge

Since for the majority of students the bioinformatics tools related to DNA sequence retrieval and analysis are unknown, on session 2 groups use different approaches, at their own pace, to get acquainted with the tools. On sessions 3–4, the instructor conducts the progress of each group providing no explicit directions on what to do, but providing feedback on students request. The instructor is active in helping students recall relevant concepts, namely of molecular biology, essentially by posing questions and suggesting related literature. As students begin to find answers to the challenge, the instructor can verify if students understand the mechanisms of nuclear receptor action.

On sessions 5–6 the instructor plays no active role, except in assuring that the groups are progressing in their assignments.

#### Session 7 (4 h): explain yourself

The final session of this activity is set for the groups to present, orally, the developed work. Presentations are concise, focusing both the biological and computational aspects of the activity, and are followed by up to 10 minutes of discussion. Individual accountability is promoted by choosing the speaker immediately before each presentation. The audience is composed by the students, the instructor and other faculty members whom had not participated as instructors.

### Educational outcome and student's feedback

This activity has been given to 240 students in 4 consecutive academic years. Most students were initially unaware of the bioinformatics tools related to DNA sequence retrieval and analysis. Like in other teaching/learning approaches [3], [4], in this activity a DNA sequence is used as the starting point. However, unlike published examples, several sequences are presented for analysis. Most groups started by exploring tutorials on the different web resources. Generally, groups have ended up identifying easily the gene to which the given sequence belonged. In contrast, the recognition of the nuclear receptor binding response element within the given DNA sequence and the identification of the corresponding modulator was a harder process. After understanding the specificities of the mechanism of hormone/vitamin mediated gene regulation, students focused attention on its relevance in cellular, tissue and all-body homeostasis. For this purpose literature databases, namely PubMed and Web of Science, became the mostly used type of bioinformatics resources.

Finally, the written reports and the oral presentations clearly revealed that all groups successfully achieved the proposed aims.

Of notice, the design for this activity promoted discussion amongst the students since, by working in groups, students had to reach consensus for the final output. In fact, the lack of fully-guided orientation (the instructor does not interfere with students' options) forces decision-making, a process that needs to be trained for the future clinical practice.

Student ratings have been gathered anonymously in the last 3 of the 4 academic years in which the activity took place. Almost all students (95%) considered that this activity should be maintained in the curriculum. On how the activity impacted on personal development, 90% of the students considered that they now understand better the usefulness of bioinformatics for their career and indicated (98%) the intention to use them in the future. As for students' opinion on integrative aspects, the feedback was very positive with 80% considering that it had much contributed to the comprehension of the Endocrine system as integrating all other body systems. Students were also given the opportunity to express their personal impression on the activity, reflecting their expectations about the activity and the drawbacks they encountered. On their own words: “(…) although the activity implied hard work I enjoyed it a lot, mainly because of the knowledge that was acquired using novel tools (…)”. Some felt that “despite the great personal evolution through the module, there are still aspects in which my evolution could have been better”, but still “recommend further bioinformatics activities functioning like this one”. The group work and the lack of imposed directions by instructors on the final content of the work was acknowledged because “it obliged us to find solutions for the problems by ourselves, which increased group communication and brought interesting discussions on different themes”. Other aspects students indicated as positive were the clinical contextualization of basic molecular mechanisms of hormonal regulation, and the improvement on skills related to literature database search.

## Discussion

This paper presents an activity that leads students to recognize, by themselves, that nuclear receptors can act as monomers or dimmers and recognize DNA binding to consensus sequences; to understand that hormones might regulate gene expression in opposite ways, by activating or inhibiting expression, and that regulation of gene expression depends on the interaction of the nuclear receptor with other transcription factors, coactivators and corepressors. When compared to other previously reported successful approaches of bioinformatics in the medical curriculum [3], [4], the activity adds the following achievements: it is an integrated rather than isolated activity, it focus on various rather than single genes and/or mutations, and it broadens the spectrum of gene regulation in health and disease, with the potential to highlight how gene expression can be a target for novel therapies.

Choosing target genes is one of the key aspects for the success of this activity. They must be clinically relevant and fulfil the following requirements: to have a hormone response element sequence in its promoter region; the hormone must effectively impact on its expression (not only having a binding sequence for the nuclear receptor) and its expression should be influenced by the interaction with other transcription factors. Table 3 lists some of such genes. For instance, the promoter of the gene encoding for corticotropin releasing hormone (CRH) contains three glucocorticoid response elements but only two of the sites can be occupied simultaneously by a glucocorticoid nuclear receptor dimmer, with site occupancy depending on other transcription factors. In addition it is a good example of a feed-back inhibition mechanism: CRH influences glucocorticoid synthesis in the adrenal glands via stimulation of the adrenocorticotropic hormone, and its synthesis is inhibited by glucocorticoids via the hypothalamus-pituitary-adrenal loop [10]. Moreover, besides its role as a hormone and its implication in disease, CRH functions as a neurotransmitter, and thus influences behaviour.

**Table 3:** Human genes and respective DNA (partial) sequence given to students.

Gene	hormone/ligand	HRE	DNA sequence (5′ to 3′)*	Physiological context	Body system involved
Corticotropin Releasing Hormone	glucocorticoids	GRE	atttttgtcaatggacaagtcataagaa	Hypothalamus-pituitary-axis regulation	Endocrine
Parathyroid Hormone	vitamin D	VDRE	aactataggttcaaagcagcacata	Bone metabolism	Skeleton and Muscle
Renin	triiodothyronine	TRE	aggtcaggtcacaatgttcct	Kidney function and blood flow regulation	Urinary; Cardiovascular
Oxytocin	estradiol	ERE	caacgcggtgaccttgaccccgg	Pregnancy and lactation; behavior	Reproductive; Central Nervous System
Prostate specific antigen	androgens	ARE	aattgcagaacagcaagtgctagctct	Male reproduction physiology; prostate cancer	Reproductive
Homeobox A4	retinoic acid	RARE	gccgaggtgaacttcaggtcagtg	Development; organogenesis of the nervous system	Peripheral and Central Nervous Systems
Muscle carnitine palmitoyltransferase	several lipids	PPRE	atgtagggaaaaggtca	General metabolism; metabolism of the cardiac muscle	Cardiovascular
NRGN/neurogranin	triiodothyronine	TRE	atggggattaaatgaggtaata	Central Nervous System development regulation	Central Nervous System
Apolipoprotein CIII	several lipids	PPRE	gcgctgggcaaaggtca	Lipid and lipoprotein metabolism	Cardiovascular
ATP binding cassette transporter G1	oxysterol	LXRE	gcaagaggtaactgtcggtcaaatcc	Lipid and cholesterol metabolism	Cardiovascular
Angiotensinogen	estradiol	ERE	tataaatagggcatcgtgacccg	Blood flow regulation; gender differences	Cardiovascular
Neuronal Serotonin receptor	glucocorticoids	GRE	tctccttgtcctttgacacgtccttta	Behavior	Central Nervous System
Insulin growth factor binding protein	glucocorticoids	GRE	attttgaacactcagctcctag	General metabolism; aging	Several
Osteocalcin	vitamin D	VDRE	ccgggtgaacgggggcatct	Bone metabolism	Skeleton and Muscle

* Each sequence contains a hormone response element (HRE) consensus sequence for the binding of the respective nuclear receptor.

In summary, by providing an activity that promotes independent search and retrieval of accurate genomic information, students develop essential bioinformatics skills and knowledge for tackling genetic problems in medicine, now and in the future.
